# Urinary Malondialdehyde (MDA) and N-Acetyl-β-D-Glucosaminidase (NAG) Associated with Exposure to Trichloroethylene (TCE) in Underground Water

**DOI:** 10.3390/toxics10060293

**Published:** 2022-05-29

**Authors:** Wen-Yu Lin, Chun-Ping Tu, Hsien-Hua Kuo, Hsien-Wen Kuo

**Affiliations:** 1 Institute of Environmental and Occupational Health Sciences, National Yang Ming Chiao Tung University, Taipei 112304, Taiwan; weyulin@epa.gov.tw (W.-Y.L.); david1989823@gmail.com (C.-P.T.); 2 Environmental Protection Administration Executive Yuan, Taipei 100006, Taiwan; 3 Nursing Department, Taipei Hospital Ministry of Health and Welfare, Taipei 242033, Taiwan; nurse20320@tph.mohw.gov.tw; 4 School of Public Health, National Defense Medical Center, Taipei 114201, Taiwan

**Keywords:** trichlorethylene (TCE), trichloroacetic acid (TCA), malondialdehyde (MDA), N-acetyl-β-D-glucosaminidase (NAG)

## Abstract

Trichloroethylene (TCE) is commonly used in various industries. If wastewater in factories is not effectively treated, the inflow into and subsequent contamination of underground water is likely. Our study assessed the association of exposure to TCE in underground water with oxidative stress and renal tubule damage. We selected 579 residents from areas with underground water contaminated with TCE. Each participant was interviewed via a questionnaire. We also assessed their urinary trichloroacetic acid (TCA) levels by gas chromatography (GC)-FID. Urinary malondialdehyde (MDA) and N-acetyl-β-D-glucosaminidase (NAG) were taken as indicators of oxidative stress and renal tubule damage. We found about 73% of the residents to have consumed underground water. The average duration of consumption was 26 years, with an average of 1.6 L per day. Currently, only 1.5% of the residents still continuously consume underground water. The consumption of underground water positively correlated with heightened urinary TCA levels (r = 0.554). Heightened urinary TCA levels, in turn, were positively associated with NAG levels (r = 0.180) but negatively associated with MDA levels (r = −0.193). The results held even after we had segmented urinary TCA levels into three groups of different levels. The elimination of the source of heightened TCE levels from various industrial effluents is essential. Residents exposed to TCE-laden underground water should periodically undergo health inspections.

## 1. Introduction

Trichloroethylene (TCE) is one of the volatile organic contaminants (VOCs) most widely found in groundwater, especially in heavily contaminated industrial sites [1]. The biodegradation of TCE under anaerobic conditions is slow, making TCE relatively persistent in subsurface waters. An Environmental Protection Agency (EPA) national groundwater survey detected TCE in approximately 10% of the wells tested [2]. Taiwan has experienced a similar problem. For example, the Radio Corporation of America (RCA) factory in Taiwan has caused serious soil and underground water contaminations at the factory site and in the nearby ground due to an improper treatment of chlorinated compounds. At that time, 252 ppb and 930 ppb of TCE contaminants were detected in well water from the factory and neighborhoods close by, respectively [3].

As TCE is a liposoluble chemical compound, long-term exposure to TCE results in its accumulation in adipose tissue, among other organs. TCE is primarily metabolized by P-450 enzymes in the liver. The metabolic reaction begins with oxidization, which creates TCE epoxides which tend to be generated through unstable chemical activation. Then, through a series of metabolic processes, two important metabolites—trichloroacetic acid (TCA) and trichloroethanol (TCE-OH)—form, along with some other metabolites [4]. In this process, approximately 18% of TCE metabolizes into TCA, and is thereafter excreted directly through urinary tract system, and another 33% metabolizes into trichloroethanol [5].

In the workplace, TCA is the main biological indicator for TCE. According to ACGIH (The American Conference of Governmental Industrial Hygienists), the permissible airborne level of TCE (i.e., TLV-TWA) in a workplace is 10 ppm. The acceptable urinary level of TCA was set as less than 100 mg/g cre. In the workplace, TCE exposure, TCA present in urine, and MDA present in blood have been consistently higher than those levels in controls. Moreover, prolonged exposure to TCE will result in significantly higher levels of MDA [6]. Because TCE increases the amount of formic acid released in urine, previous studies [7,8] have determined it to be a renal toxin and indicated that both acute exposure to high amounts of TCE and long-term but gradual exposure to TCE harm renal tubule. The mechanism of kidney damage has been explained by renal tubular cell necroptosis using trichloroethylene hypersensitivity syndrome mice [9]. N-acetyl-glucosaminedase (NAG) is already known as an early indicator for renal tubule damage and can be used as an early diagnosis tool [10]. The mechanism by which TCE harms the human renal tubule is still unclear. The possible mechanism might be related to the production of free radicals within the body, leading to oxidative damage [11]. For our study, we chose the vicinity of an industrial park in central Taiwan as our target area of research—specifically those areas in which citizens might have been exposed to underground water present with TCE. Our topic of interest was to measure the relationship between urinary TCA levels with MDA and NAG levels in urine.

## 2. Materials and Methods

### 2.1. Polluted Areas and Their Citizens

The polluted area in question was the export processing area of Tanzi industrial park in Taichung City. Established by the government in 1969, this area occupies 26.1 hectares that are mainly used for the production of electronic components. However, this area also contains businesses in the refinery industry, optics, wholesale, and other types of manufacturing. In the past, some factories in this area received fines due to the improper wastewater disposal, which polluted underground water within the vicinity of residences. Our study focuses on this area that includes the residents of neighborhoods that draw from 17 polluted wells (as reported by Taichung’s government’s EPA), an area in which 579 people reside. By engaging the cooperation of the heads of villages and neighborhoods, we used a door-to-door interview process for every resident, allowing us to find citizens who had been affected by the contamination in underground water. The total population of 563 residents was enrolled and interviewed by questionnaire. The contents of the questionnaire included demographic information (age, gender, marital status and education level, how long they had lived live there (years), how long they had used the tap and underground water (years) and whether they used water-purification systems). However, due to time limitations and personal willingness, only 180 residents provided spot urine to determine the biological monitored of TCE (Urine samples).

### 2.2. Determination of Urinary TCA, MDA and NAG Levels

We modified an analytical method of urinary TCA from that of Kuo et al. [12] and Wu et al. [13]. We used GC with an FID detector. The calibration curve of TCA ranged from 0.1 μg/mL to 100 μg/mL. The coefficient of correlation was 0.9993. The coefficients of variation (CV) in within- and between-group reproducibility were 0.01% and 0.07%, respectively. The analytical method of urinary MDA was a modified method from Wong et al. [14] using HPLC/UV (1260 infinity, G1311C, 1260 Quat pump, Agilent, U.S.A.). The detection limit was 0.04 μmol/L. Good reproducibility of MDA was less than 7%. The average recoveries were 100.3%, 83.7%, and 84.8%, with spikes at 80 ppb, 800 ppb, and 2 ppm of MDA. We used a commercial kit for measuring urinary NAG levels (Diazyme laboratories, Poway, CA 92064, USA). A stability test was obtained using CV with 1.02%, 0.25%, and 2.55% in three NAG concentrations.

### 2.3. Statistical Analysis

We used SPSS17 to perform statistical analyses on the data collected in the questionnaires as well as the levels of TCA, MDA, and NAG in urine. Pearson’s correlations examined the associations between the experience of using underground water and TCA, MDA, and NAG levels in urine. Univariate analysis tested urinary TCA’s effects on MDA and NAG levels. Over 75% of the participants showed MDA > 0.32 mg/g cre. and NAG > 12 U/g cre. Multiple logistic regression was used to examine the effects of urinary TCA levels (low, medium, or high) on MDA and NAG levels.

## 3. Results

Table 1 shows demographic information and information regarding the use of underground water obtained using the questionnaire in the total sampled population and urine samples. Due to time restrictions and personal willingness, only 180 residents from the total sampled population simultaneously provided a urine sample and a completed questionnaire. Demographic information was similar in the two groups. Male and females were about equally represented in the two groups. Regarding the age distribution, the most populous age range was that from 30 to 65 years old, occupying 65% of the total population and that providing urine samples. Those over 65 accounted for 23.1% and 22.2% of the total population and the population providing urine samples, respectively. Those who had lived for 5–30 years in the polluted area accounted for 57.6% and 54.6% of the total population and the population providing urine samples, whereas those who had resided there for over 30 years accounted for 34% and 37.4% of the population in the two groups, respectively. The average length of residence was 27.1 years. Currently, over 90% of the total population and the population providing urine samples use tap water, and 3% use underground water. Those who had used underground water in this way for more than 30 years accounted for 31.4% and 40.7% of the population in the two groups, respectively. The average lengths of underground-water-as-normal-water usage were 26.5 and 29.3 years in the two groups, respectively. Residences using water-purification systems accounted for 60.1% and 62.9% of the population in the two groups, respectively.

Table 2 shows the correlation analysis on urinary TCA, MDA (our indicator for lipid peroxidation), NAG (our indicator for kidney functioning), and creatinine levels. Urinary TCA levels (mg/g cre), after being corrected for creatinine levels, showed a significant, negative correlation with MDA (r = −0.193, *p* < 0.05). We found the daily consumption of underground water to be positively correlated with urinary TCA levels (μg/mL) (r = 0.554, *p* < 0.01). Yet, the significant correlation between TCA (mg/g cre) and NAG levels was positive (r = 0.180, *p* < 0.05). MDA and NAG showed a significant negative correlation (r = −0.155, *p* < 0.05). Urinary creatinine and NAG levels also had a significant negative correlation (r = −0.343, *p* < 0.01).

Table 3 divides urinary TCA into high, medium, and low levels. The ANOVA results for these levels and their relationships with MDA and NAG levels are displayed. One apparent result is that as the TCA level increases in the urine, the NAG level decreases. Analysis of variance (ANVOA) for these three levels differs significantly. A contrasting trend is that between the urinary TCA level and its NAG counterpart: they are directly proportional. Yet, again, ANOVA significantly differs by TCA level.

Table 4 shows the TCA density levels with cutoff points for MDA and NAG levels (>5μmol/L and >12 U/g cre., respectively); this is a multivariate logistic regression adjusted for sex and age. Using a urinary TCA level below 3.59 mg/g cre. as a reference point shows a lower odds (OR) ratio for an MDA level above 0.32 mg/g cre., but without statistical significance. With a TCA cutoff point of 12 U/g cre., the OR of the mid-level group (3.59 mg/g cre. to 10 mg/g cre.) to the low-level group (<3.59 mg/g cre.) is 4.08 (*p* = 0.017). Additionally, the corresponding OR for the high-level group (>10 mg/g cre.) is 3.05, a marginally significant result (*p* = 0.066).

## 4. Discussion

Our study looked at the communities in the vicinity of 17 wells that were deemed by our government’s EPA as polluted. In 2010, the EPA tested these wells, finding five as having TCE levels exceeding the accepted level for water use (0.05 mg/L), at 0.0779, 0.0551, 0.0845, 0.0712, and 0.155 mg/L. However, neither data on TCE levels in underground water used in resident’s homes in the past nor data on the current TCE levels of underground water have been available until our study. For the purpose of collecting these data, we focused on neighborhoods within the vicinity of the export processing area. Factories in this area previously exposed the environment to wastewater by directly dumping it into irrigation channels or wells, which led to the pollution of 10 square kilometers of water in the surrounding area. The residents of this area, without their own water supply system, relied on underground water as their main water source. Over a long time period, such an action could have had an impact on the residents’ health. Only in 2010 did the residents begin using tap water.

The results of our study show that the citizens consuming water contaminated with TCE were likely exposed to low levels, as the average level of their urinary TCA was 7.39 µg/L or 9.83 mg/g cre., which is lower than the Biological Exposure Indices (BEI) standard for workers (100 mg/g cre.). Using Taiwan’s acceptable exposure level (PEL) of air TCE in the workplace, which is 50 ppm (269 mg/m^3^), for this calculation, we found that the average laborer was exposed to—at most—216.0 mg per day, a number that far exceeds the TCE exposure found in residents drinking the contaminated water. Nevertheless, we found a positively significant correlation between the daily consumption of underground water and TCA levels in urine (r = 0.554, *p* < 0.01) (data not shown), meaning that the long-term consumption of TCE in underground water contributes to urinary TCA levels. 

Ikeda et al. [15] recommend the monitoring urinary TCA to estimate air exposure to TCE. Souček and Vlachova [16] found a positive correlation between the intensity of TCE exposure and TCA levels in urine, though the amount of TCA formed from retained TCE appears to vary with the species tested. The authors indicated that the concentration of the TCA in the urine reaches its maximum between 24 and 48 h, subsequently decreasing exponentially for a few days. A study in Finland found 212 μg/L TCE and 180 μg/L perchloroethylene (PCE) in underground water. The results show the average urinary TCA levels to be 19 μg/L (<1–110 μg/L) and 7.9 μg/L (<1–50 μg/L) higher than those of the control groups, who showed average TCA levels of 2.0 μg/L (<1–6.4 μg/L) and 4.0 μg/L (<1–13 μg/L), respectively [17]. Comparing the results with ours, which were 7.39 μg/L and 9.83 mg/g cre., we find that they are similar to our findings. However, the current study found that the largest TCA levels were 136.50 μg/L and 167.57 mg/g cre. among residents who had ingested polluted water, which is higher than that in the average concentrations in US citizens. For the US counterparts, the average urinary TCA level was 2.9 μg/L, and the 25th, 50th, and 90th percentiles were 0.6 μg/L, 3.3 μg/L, and 23 μg/L, respectively [18]. Even though residents living in this polluted region have mostly changed to using tap water, some people with high urinary TCA concentrations may continuously be exposed to polluted underground water through dermal absorption. It is crucial to persuade residents to discontinue using underground water in polluted region, even they do not orally intake it directly.

After TCE metabolizes into TCA in the liver, its half-life in the human body is two to six days [19]. In humans, it is metabolized quickly; if a person does not drink or use polluted groundwater, the metabolized TCA in the urine will quickly be excreted and there will be a fall in levels. In our study, we found the daily consumption of underground water to be positively correlated with urinary TCA levels (μg/mL) (r = 0.554, *p* < 0.01). Since most participants stopped using the contaminated groundwater in 2010, switching instead to tap water, this resulted in reasonably low urinary TCA levels, but might also have resulted in an underestimation of the past exposure to TCE in groundwater. Another factor possibly overlooked is the chance that the TCA found in urine came from PCE and not TCE exposure. 

Two major TCE metabolites, chloral hydrate (CH) and TCA, as well as a reported minor metabolite, dichloroacetic acid (DCA), also produce liver tumors in mice [20,21]. TCA and DCA have been shown to cause oxidative stress in mice. However, both increase the formation of thiobarbituric acid reactive substances (TBARS) in a dose–response manner in mouse liver following a single oral dose, suggesting that each was capable of yielding a radical species which could initiate lipid peroxidation [22]. However, our results show the opposite. As TCA levels in urine rose, MDA in the urine fell significantly; urinary TCA and MDA showed a negative correlation. This is explained by the fact that urinary MDA level is an oxidative stress indicator, but it lacks specificity and is affected by many intrinsic and extrinsic factors. In addition, over 90% of residents living in the polluted region have not continuously used underground water for a long period, and previously consumed TCE from underground water is quickly metabolized and excreted in urine. Due to the short biological half-life (2.1 to 6.3 days) of TCA in urine for most of the currently consumed TCE-polluted underground water, TCA in urine samples mostly represents recent exposure [13]. Consequently, the number of subjects exposed to TCE levels from consumption of underground water in our study was considerably low, with high individual variation, making a reverse relationship between TCA and MDA in urine harder to verify.

In contrast to previous studies, which included subjects exposed to high levels of TCE over short periods, our study focused on subjects exposed to low levels of TCE over a long period. An animal study from Channel et al. [23] showed a significant increase in cell and peroxisomal proliferation in the high-level group (at 1200 mg/kg/day). Urinary TCA levels due to exposure to TCE quickly diminish because TCE’s half-life ranges from 2.1 to 6.3 days [17]. Similarly, an animal study indicated a marked formic aciduria following TCE which, after 8 weeks’ exposure, did not produce renal injury. Only the increase in NAG suggested that renal damage may occur following longer exposure, while inconsistent findings were found for other renal indicators [24]. However, our study participants with renal dysfunction (NAG > 12 U/g cre.) had higher levels of TCA and MDA than those who had normal renal function. This result is consistent with the increasing number of findings linking oxidative stress to patients with chronic kidney disease (CKD). Patients with renal impairments may have increased oxidant activity and a reduced antioxidant capacity, and this is increased in a graded manner with increasing renal dysfunction [25]. A study on painters who had worked for more than ten years supported the hypothesized association between solvent exposure and the development of chronic renal failure [26]. However, another study of a small group of male metal degreasers in Sweden observed no increase in NAG excretion into the urine, concluding that TCE was not nephrotoxic at low exposure levels [27]. 

Several studies have linked exposure to TCE to NAG. Mensing et al. [8] indicated significantly increased concentrations of NAG in the urine of rats exposed to 500 ppm TCE for long time periods. Wang’s study [9] confirmed that TCE sensitization caused damage to renal tubules and renal tubular epithelial cell (RTEC) necroptosis in an animal study. However, comet assays did not detect DNA-strand breaks in kidney cells or histological alterations in glomeruli and tubuli. Metal degreasers in central Sweden exposed to high levels of TCE (>250 ppm) showed urinary NAG levels of 5.27 U/g cre. in a cross-sectional study. Green et al. [28] suggested that kidney damage could occur at exposure concentrations higher than those encountered in this study. Seldén et al. [27] indicated a weak positive correlation (r = 0.48; *p* < 0.01) between NAG activity and the concentration of urinary TCA. The authors concluded that TCE does not seem to be nephrotoxic at low exposure levels, a conclusion similar to the findings of Mensing et al. [29], which showed that both acute exposure to high amounts of TCE and long-term but slow exposure to TCE can damage the renal tubule. The potential mechanism was explained by reactive trichloroethene (TCE) metabolites and oxidative stress being involved in TCE-mediated autoimmunity. Significantly increased serum MDA-protein adducts were revealed using an animal study [30], which demonstrated CYP2E1-mediated TCE metabolism in autoimmune response and an important role of the Nrf2 pathway in TCE-mediated autoimmunity. However, most previous studies on TCE have investigated occupational workers, and they have tended to be studies on high TCE levels. Our study, in contrast, looked at lower levels of TCA in the urine and TCA’s relationship with NAG levels for a general population exposed to underground water pollution; we found a statistically significant positive correlation (r = 0.180). Although our study’s TCA levels were within the BEI range (100 mg/g cre.), their changes were clearly dose-dependent and consistent with one of the proposed mechanisms of trichloroethylene-induced kidney toxicity. 

Our study was not without its limitations. First, our cross-sectional study was limited in its ability to establish causal relationships between exposure to TCE and oxidative stress and renal dysfunction. Second, despite knowing the location of the contamination, we did not examine the actual TCE levels in the underground water. Third, only 180 residents participated or donated urine specimens. Reasons included moves/transfers, lack of time to interview, refusal, and an inability to reach some residents; these reasons are unlikely to have affected our study’s purpose or outcome. In addition, Table 1 does not indicate significance for demographic information and experience using tap and underground water in the total population and the population providing urine samples. These limitations might result in an underestimation in our findings. Since urinary TCA may be quickly metabolized and excreted due to its short-half life in urine, our results indicate low TCA levels with high variation in the study population. Consequently, we present revised relationship between TCA and MDA in urine. Our study did not consider the effect of metabolic enzyme gene polymorphisms in TCE exposure [31]. We assumed the existence underestimated findings from information bias due to nondifferential misclassification. However, renal damage could possibly occur from consecutive exposures to low TCE concentrations in underground water. Thus, for those not working in industries that risk exposure to TCE but are still continuously exposed to polluted underground water through dermal exposure, it is possible to indicate that the past use of underground polluted water has contributed to adverse renal effects among residents. Therefore, it is crucial to immediately remediate TCE-contaminated groundwater under environmentally-related conditions using the continuous supplement of high concentrations of KMnO4 [32] and biomimetic iron-nitrogen-doped carbon [33].

## 5. Conclusions

We found the past use of underground water to be positively correlated with urinary TCA levels. Urinary TCA levels were, in turn, positively associated with NAG levels but negatively associated with MDA levels. Given the frequent use of TCE as a degreasing solvent, plus its recalcitrance and chemical properties, this environmental pollution in underground water is universal. While tap water has provided in the polluted area, Taiwan’s EPA encouraged residents to avoid continuously using underground water in daily life before it was mitigated. Due to the adverse health effects on the kidneys to which our findings allude, we recommend eliminating the source of heightened TCE levels from various industrial effluents. In addition, residents should periodically monitor their health effects if they have been exposed to TCE in underground water.

## Figures and Tables

**Table 1:** Demographic information and information regarding the use of underground water obtained using a questionnaire in the total population and urine samples.

	Total Population (N = 563) n (%)	Urine Sample (N = 174) n (%)
Sex	(n = 563)	(n = 174)
Male	288 (51.2)	93 (53.4)
Female	275 (48.8)	81 (46.6)
Age (year)	(n = 558)	(n = 171)
<30	65 (11.6)	21 (12.3)
30–65	364 (65.2)	112 (65.5)
>65	129 (23.1)	38 (22.2)
Mean	52.10 ± 16.85	52.11 ± 16.9
Duration of living (year)	(n = 538)	(n = 163)
<5	45 (8.4)	13 (8.0)
5–30	310 (57.6)	89 (54.6)
>30	183 (34.0)	61 (37.4)
Mean	27.12 ± 17.36	29.20 ± 18.42
Current sources of drinking water	(n = 573)	(n = 171)
Pape water	522 (91.1)	160 (93.5)
Buy water	13 (2.3)	6 (3.5)
Underground water	17 (3.0)	3 (1.8)
unknown	21 (3.7)	2 (1.2)
Duration of sources of drinking water (year)	(n = 538)	(n = 171)
<3	358 (66.5)	112 (65.5)
3–10	55 (10.2)	21 ( (12.2)
>10	125 (23.2)	38 (22.3)
Past use underground water (n = 531)	451 (84.9)	120 (80.0)
Duration of use underground water (year)	(n = 449)	(n = 140)
<10	56 (12.5)	12 (8.6)
10–30	252 (56.1)	71 (50.7)
>30	141 (31.4)	57 (40.7)
Mean	26.5 ± 15.0	29.3 ± 17.9
Used water-purification systems	324 (60.1)	100 (62.9)

**Table 2:** Average concentrations of creatinine, TCA with MDA and NAG and correlation matrix (N = 180).

	Creatinine (mg/dL)	TCA (mg/g cre.)	MDA (mg/g cre.)	NAG (U/g cre.)	Mean ± SD
Creatinine (mg/dL)	1.000	−0.298 **	0.560 **	−0.343 **	114.16 ± 66.61
TCA (mg/g cre.)	-	1.000	−0.193 *	0.180 *	9.83 ± 18.91
MDA (mg/g cre.)	-	-	1.000	−0.155 *	0.15 ± 0.10
NAG (U/g cre.)	-	-	-	1.000	7.07 ± 9.32

* *p* < 0.05 ** *p* < 0.01.

**Table 3:** Univariate analysis on urinary TCA affecting MDA and NAG levels.

	MDA (N = 179) (mg/g cre.)			NAG (N = 161) (U/g cre.)		
	n	mean ± SD	*p*	n	mean ± SD	*p*
TCA (mg/g cre.)						
<3.59	92	0.17 ± 0.09	0.002	86	5.41 ± 6.28	0.039
3.59–10	42	0.13 ± 0.09		38	8.14 ± 7.64	
>10	42	0.11 ± 0.12		37	9.83 ± 14.74	

**Table 4:** Urinary TCA levels correlated with levels of MDA and NAG using multiple logistic regression adjusted for gender and age.

	MDA > 0.32 mg/g cre.				NAG > 12 U/g cre.			
	n	OR	95%CI	*p*	n	OR	95%CI	*p*
TCA (mg/g cre.)								
<3.59	90	1			83	1		
3.59–10	37	0.54	0.10–2.85	0.469	33	4.08	1.28–12.94	0.017
>10	36	0.24	0.03–2.03	0.188	31	3.05	0.93–10.02	0.066

## Data Availability

Not applicable.
